# Assessment of flavivirus RNA stability and infectivity in various water environments

**DOI:** 10.1186/s41182-025-00686-9

**Published:** 2025-01-24

**Authors:** Yuka Sano, Hawraa Al-Alawi, Misao Himeno, Ryuichi Majima, Kazumi Haga, Myo Thura Kyaw, Satoshi Taniguchi, Meng Ling Moi

**Affiliations:** 1 School of International Health, Graduate School of Medicine, the University of Tokyo, Tokyo, 113-0033 Japan; 2 Department of Biology, College of Science, Sultan Qaboos University, Muscat, Oman

**Keywords:** Orthoflavivirus, Dengue, Zika, Japanese encephalitis, Yellow fever, Surveillance, RNA virus, Environment, Water

## Abstract

**Introduction:**

Flaviviruses such as dengue virus (DENV), Zika virus (ZIKV), Japanese encephalitis virus (JEV), and Yellow fever virus (YFV) are mosquito-borne RNA viruses causing major public health threats in major parts of the world. While DENV and ZIKV have been detected in urine samples, data on the presence and stability of flaviviruses in the water environment are limited.

**Methods:**

In this study, we determined the stability and infectivity of flavivirus in different water environments by utilizing RT-qPCR and plaque assay to explore the feasibility of environmental detection and surveillance of flaviviruses.

**Results:**

Viral RNA could be detected for up to 49-days, at 4 °C, 25 °C and 37 °C temperatures, and infectious particles could be detected for up to 7 days. While our findings showed that flaviviral RNA has higher stability and better detection rates at lower temperatures, the infectious capacity of flaviviruses was comparatively short at 7 days.

**Conclusions:**

Our results indicate that flaviviruses retains limited infectivity in general water environments and highlight the feasibility of detection and surveillance in various epidemiologic and environmental settings.

**Supplementary Information:**

The online version contains supplementary material available at 10.1186/s41182-025-00686-9.

## Background

The Orthoflavivirus genus of a single stranded RNA viruses including dengue virus (DENV), Zika virus (ZIKV), Japanese encephalitis virus (JEV), and Yellow fever virus (YFV) that are transmitted by vectors, commonly mosquitoes. These viruses pose a significant public threat globally [1–6]. According to the World Health Organization (WHO), DENV is the most common arboviral disease globally but persisting as a major concern in tropical and subtropical regions [7, 8]. For instance, the Culex mosquitoes transmit JEV which is also endemic in Eastern, Southern Asia and the Pacific Rim [5]. The Yellow fever is endemic in tropical areas such as South America and Africa [3]; whereas, ZIKV is endemic in the Americas, Asia, several Pacific islands, and Africa [4, 9]. DENV, YFV, and ZIKV have the potential to re-emerge and spread worldwide to new geographical area where the *Aedes* mosquitoes are present. For instance, in Oman, the presence of DENV vector has been documented since 2008. Yet, the first autochthonic dengue outbreak was reported in 2018 and re-emerged in 2022. This represented a shift in disease spreading dynamics altered by climate change, globalization, and vector proliferation [10]. Thus, comprehensive surveillance of flaviviruses across the region is important in monitoring transmission and emergence of flaviviruses in the region.

Recent studies have investigated the stability of SARS-CoV-2 and the study findings suggest that SARS-CoV-2 is highly stable in various conditions [12, 13]. In SARS-CoV-2 surveillance, water environment and sewage surveillance have emerged as an important tool for monitoring COVID-19 epidemiological trends. Additionally, in a study conducted previously has demonstrated that ZIKV is structurally more stable as compared to DENV at various temperature [11]. Furthermore ZIKV remained infectious, but the infectivity of DENV-2 and DENV-4 strains decreased significantly at high temperatures. To date, there is limited data on the stability of flaviviruses in water environments. This study aims to determine the stability and infectivity of flaviviruses at different temperatures and water environments. Here, we determined the infectivity and RNA stability of flaviviruses in various water environments by using quantitative real-time RT-PCR (RT-qPCR) and conventional plaque titration methods up to 49 days.

## Methods

### Viruses and cell lines

Vero 9013 cells (African green monkey kidney epithelial cells) was used for plaque assay. Vero cells were cultured in Eagle’s Minimum Essential Medium (EMEM) (FUJIFILM Wako Pure Chemical Corporation, Osaka, Japan), supplemented with heat-inactivated 10% fetal bovine serum (FBS) (NICHIREI BIOSCIENCES INC., Tokyo, Japan). The cells were maintained at 37 °C in 5% CO_2_ [14]. DENV-1 01–44-1 HuNIID strain (GenBank accession no. AB111070), JEV OH0566 strain (GenBank accession no. AY508813), YFV YFV 17D strain (GenBank accession no. MT107250.1)) and ZIKV PRVABC59 strain (GenBank accession no. KU501215) were propagated on Vero cell at 37 °C in 5% CO_2_ for up to 4–5 days until CPE formation were observed [12]. Virus titers were determined by plaque assay as plaque-forming units per milliliter (PFU/mL).

### Sample processing

In this study, tap, well, river, and sea water samples were obtained from various locations in Tokyo, Japan (Supplementary Table 1). DENV-1, JEV, YFV and ZIKV were spiked into the four water environment samples or EMEM as a control with final virus concentration of 1 × 10^5^ PFU/mL. Well, river and sea water were filtered to avoid contamination with 0.45 µm PVDF. The inoculated water environment samples were maintained in separate temperatures. First, DENV-1, JEV, YFV and ZIKV spiked samples were stored at 4 °C and 25 °C, and collected at Day 0, Day 4 and Day 7. In addition, the range of the experiment was extended for DENV-1, five types of samples were spiked and stored at 4 °C and 25 °C until Day 49. In addition, river water and EMEM were spiked with DENV-1 and collected from Day 0 to Day 49 at 9 incubation period points at 37 °C.

### Plaque assay

Plaque assay was performed to assess the infectivity of the flaviviruses [15]. The samples were first diluted 1:10–1:100 with the EMEM/10%FBS. A total of 100 µL of diluted sample was inoculated onto cells in a 12-well plate, and the plate was incubated at 37 °C in 5% CO_2_ for 60 min. Then, 2 mL of maintenance medium (1% methylcellulose with EMEM 2% FBS) was added to each well. Upon the development of plaques at 4–5 days after incubation, the cells were fixed with neutral formalin (0.3%) and stained with methylene blue solution to determine the plaque-forming units. Plaque counting was performed visually, and viral titer in each sample was defined as plaque-forming units per milliliter.

### Taq-Man real-time PCR

The number of viral genome copies in samples was determined by RT-qPCR [16]. The primers were designed and prepared, followed by cascaded steps using several kits with its prescribed protocol including; Quick Viral RNA kit (Zymo research, CA, USA), PrimeScript One Step RT-PCR Kit Ver. 2 (Takara Bio Inc, Shiga, Japan), Nucleospin Gel and PCR Clean-up kit (Takara Bio Inc, Shiga, Japan), T7 RiboMAX™ Large Scale RNA Production System (Promega, Madison, WI, USA) and RNA Clean & Concentration-25 kit (Zymo research, CA, USA). Viral RNA was extracted from 150 μl of each virus spiked samples by using Quick Viral RNA kit, following the manufacturer’s protocol. Purified RNA was stored at − 80 °C until RT-qPCR test. The sequences of used primers and probe on DENV-1, JEV, ZIKV and YFV of NS3 for RT-qPCR were previously described [16–18]. For RT-qPCR, 5 μl of RNA was mixed with 5 µL of TaqMan Fast Virus 1-Step Master Mix (Applied Biosystems, Waltham, MA, USA), 0.25 µL of 100 µM gene-specific forward and reverse primers, 0.5 µL of 10 µM gene-specific probe and 9 µL nuclease-free water to a final volume of 20 μl of reaction mix (Taq-Man Fast Virus 1-Step Master Mix kit, Life Technologies, USA). The RT-qPCR cycle conditions were set as follows: RT-qPCR step for 5 min at 50 °C, 1 cycle; 20 s at 95 °C 1 cycle; 3 s at 95 °C and 30 s at 60 °C, 40 cycles. Data were collected and analyzed after 40 cycles.

### Statistical analysis

Analyses of data were performed in GraphPad Prism version 10.2.3. Plaque titration and RT-qPCR assays were tested in triplicates and the study outcomes were compared using two-way ANOVA with multiple comparisons, Sidak test. *P*-value of less than 0.05 (*P* < 0.05) is considered statistically significant. The half-life time (t1/2) of infectious virus in each sample was calculated using the following equation. t 1/2 = loge2/k, k = decay constant [19]. Decrease rate was determined as below. Reduction rate (%) = [(EMEM value–sample value)/EMEM value] × 100. If the decrease rate was negative, it was adjusted to 0%.

## Results

**Fig. 1:**
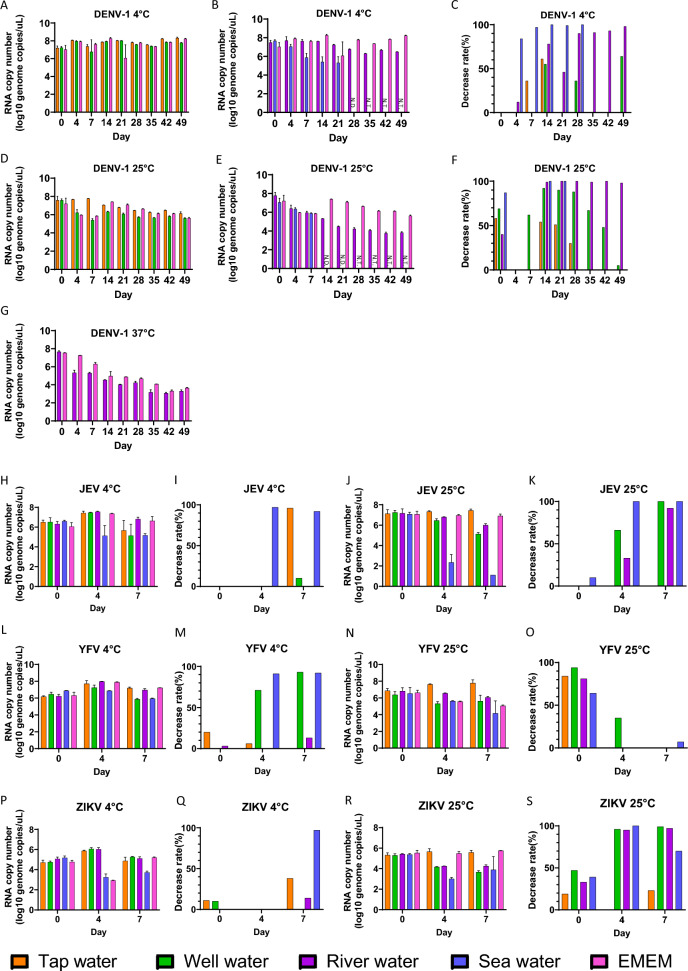
Viral RNA genome stability of DENV-1, JEV, YFV and ZIKV in aquatic waters and EMEM for each temperature. Viral genome copy number in each specimen is shown as mean ± standard deviation log10 genome copies/μl. RT-qPCR assays were performed in triplicate. **A**, **B**, **D**, **E**, **G** Viral RNA copy number of DENV-1 in aquatic waters and EMEM. **C**, **F** Decrease rate of DENV-1 against EMEM in aquatic waters. **H**, **J** Viral RNA copy number of JEV in aquatic waters and EMEM. **I**, **K** Decrease rate of JEV against EMEM in aquatic waters. **L**, **N** Viral RNA copy number of YFV in aquatic waters and EMEM. **M**, **O** Decrease rate of YFV against EMEM in aquatic waters. **P**, **R** Viral RNA copy number of ZIKV in aquatic waters and EMEM. **Q**, **S** Decrease rate of ZIKV against EMEM in aquatic waters. N.D. indicates not detected. N.T. indicates not tested

In this study, four different conditions were tested by using the following flaviviruses, DENV-1, JEV, YFV and ZIKV. Each flavivirus was spiked and collected at 4 °C and 25 °C across 3 incubation time points: Day 0, Day 4 and Day 7. Given the current rise in the reported cases of dengue virus and to investigate its dynamics, the experiment for DENV-1 had been modified to include an extended testing period at 4 °C and 25 °C to Day 49. Along with testing the DENV-1 in river water at 37 °C for Day 49 to mimic the river temperature in the tropical region, in which flaviviruses actively circulates. Viral RNA and infectious virus were determined by RT-qPCR and plaque assay, respectively. As shown in Fig. 1, the viral RNA levels remained constant at cooler environments except for sea water. At 25 °C, high RNA degradation rate was detected over time. However, in the neutral osmotic environment (i.e., tap water) viral RNA was comparatively stable at 25 °C. Furthermore, the RNA degradation rate in DENV-1 was found to increase over time at 37 °C. DENV-1 were not detected from Day 28 onwards at 4 °C in sea water samples. At 25 °C, DENV-1 was not detected from Day 14 onwards in sea water samples. Decrease rate relative to EMEM is also shown in Fig. 1. The decrease rates in sea water were high for all viruses at both 4 °C and 25 °C. For DENV-1, which had an extended testing period, an increased decrease rate in river water was observed at 4 °C and 25 °C. When comparing Day 0 and Day 7, there was a significant reduction in viral RNA of DENV-1 at 37 °C in river water (*P* = 0.0022) and EMEM (*P* = 0.0091), YFV at 4 °C in sea water (*P* = 0.0390), and ZIKV at 4 °C in sea water (*P* = 0.0385). In contrast, no significant differences were observed in other water environments at 4 °C, 25 °C and 37 °C, respectively (*P* > 0.05).

**Fig. 2:**
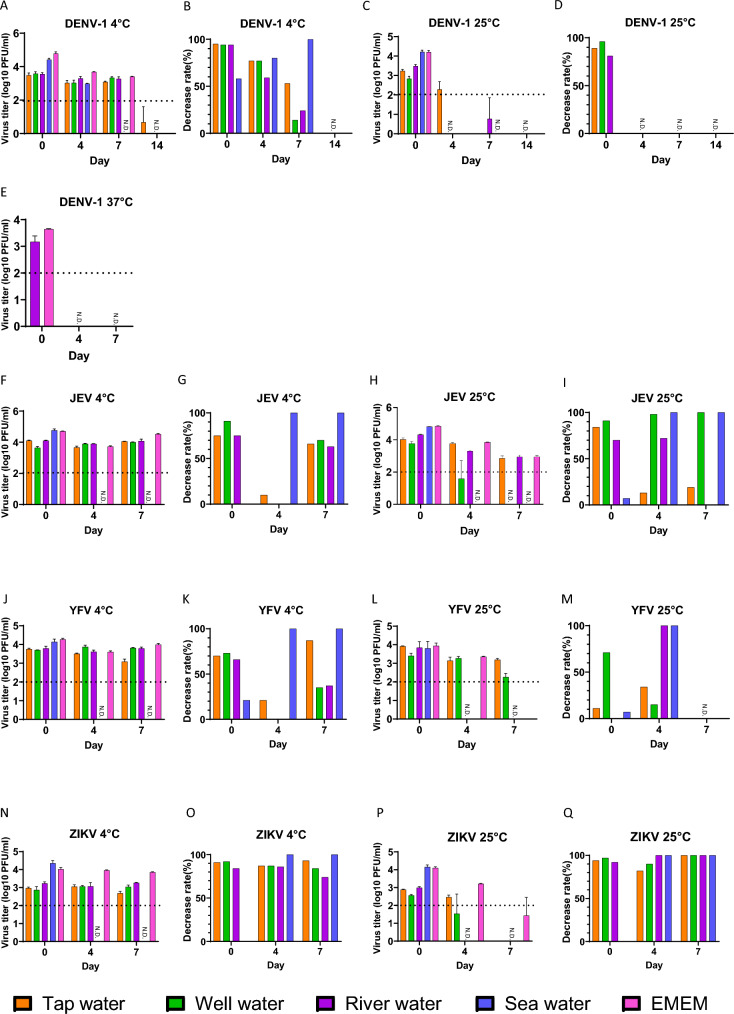
Viral infectivity of DENV-1, JEV, YFV and ZIKV in aquatic waters and EMEM for each temperature. Viral titers are shown as mean ± standard deviation log10 PFU/ml. Plaque assays were performed in a triplicate. Dash line indicates detection limits (1.0 × 10^2^ PFU/ml). Some bars below detection limit without N.D. labels mean virus was detected in several wells but not all wells in the triplex wells. **A**, **C**, **E** Virus titer of DENV-1 in aquatic waters and EMEM. **B**, **D** Decrease rate of DENV-1 against EMEM in aquatic waters. **F**, **H** Virus titer of JEV in aquatic waters and EMEM. **G**, **I** Decrease rate of JEV against EMEM in aquatic waters. **J**, **L** Virus titer of YFV in aquatic waters and EMEM. **K**, **M** Decrease rate of YFV against EMEM in aquatic waters. **N**, **P** Virus titer of ZIKV in aquatic waters and EMEM. **O**, **Q** Decrease rate of ZIKV against EMEM in aquatic waters. N.D. indicates not detected. N.T. indicates not tested

All flavivirus demonstrated a similar trend in infectivity at 4 °C as determined by plaque assay (Fig. 2). In contrast, at 25 °C the flaviviruses exhibited fluctuations in virus infectivity and the steepest decline occurred in sea water, at both 4 °C and 25 °C. At cooler environments, with the exception for sea water, the infectivity rate of all viruses remained stable through the testing period up to Day 7. DENV-1 was not detected in all samples, with the exception for tap water, from Day 14 onwards at 4 °C. Similarly, DENV-1 was not detected from Day 4 onwards at 25 °C. The decrease rates in sea water increased for all viruses at both 4 °C and 25 °C. The decrease rates of ZIKV in all samples were 100% at 25 °C. When comparing Day 0 and Day 7, there was a notable decrease in virus infectivity of DENV-1 at 4 °C and 25 °C in both sea water (4 °C *P* = 0.0015, 25 °C *P* < 0.0001) and EMEM (4 °C *P* < 0.0001, 25 °C *P* < 0.0001), and at 37 °C in river water (*P* = 0.0437) and EMEM (*P* = 0.0015). In addition, there was a significant reduction in infectivity of JEV at 4 °C in both sea water (*P* < 0.0001) and EMEM (*P* = 0.0204), and at 25 °C in tap (*P* = 0.0408), river (*P* = 0.0004), sea water (*P* < 0.0001) and EMEM (*P* < 0.0001). Furthermore, there was a notable decrease in infectivity of YFV at 4 °C in both sea water (*P* < 0.0001) and EMEM (*P* = 0.0036). Finally, there was also a reduction in infectivity of ZIKV at 4 °C in sea water (*P* < 0.0001), and at 25 °C in both sea water (*P* < 0.0001) and EMEM (*P* < 0.0001). In contrast, no significant differences were observed in other aquatic environments at 4 °C and 25 °C, respectively (*P* > 0.05). The correlation coefficient was analyzed in data between viral RNA and infectivity. The correlation coefficient of DENV-1, JEV, YFV, ZIKV were 0.73, 0.71, 0.49 and 0.53, respectively. Half-life values (Day) of DENV-1, JEV, YFV and ZIKV were calculated, the results indicate all four viruses had longer half-lives at 4 °C than at 25 °C (Table 1).

**Table 1:** Half-life value (day) of DENV-1, JEV, YFV and ZIKV in aquatic waters and EMEM for each temperature

Virus	Sample	Half-life value (day)	
		4 °C	25 °C
DENV-1	Tap water	5.9	8.0
	Well water	69.7	N.T
	River water	59.0	N.T
	Sea water	7.1	N.T
	EMEM	14.2	N.T
JEV	Tap water	395.4	13.8
	Well water	N.D	3.2
	River water	660.5	12.5
	Sea water	N.T	N.T
	EMEM	124.2	9.7
YFV	Tap water	24.6	23.5
	Well water	N.D	11.9
	River water	N.D	N.T
	Sea water	N.T	N.T
	EMEM	71.5	17.1
ZIKV	Tap water	47.2	17.6
	Well water	N.D	5.4
	River water	N.D	N.T
	Sea water	N.T	N.T
	EMEM	112.3	4.6

N.D. indicates not determined. The test results were below the detection limit of the assay done

N.T. indicates not tested

## Discussion

This study examines the stability and infectivity of flaviviruses in different aquatic environments to determine viral survival capability in association with environmental factors. A number of studies have highlighted the importance of investigating RNA viruses in aquatic environments. Viral RNA stability in seawater has been studied that RT-PCR detection revealed the presence of intact virus particles despite the rapid RNA degradation in unfiltered seawater [20]. While the traditional transmission mode of terrestrial orthoflaviviruses is through arthropod vectors, recent findings suggest the presence of flaviviruses in both marine and freshwater environments. In this context, the West Nile virus (WNV), which also belongs to the Flaviviridae family, has been detected in marine hosts such as killer whale (*Orcinus orca*) and harbor seal (*Phoca vitulina*) with a potential of crossover taking place [21]. Additionally, JEV has also been detected in a symptomatic earless seal in Japan [22]. While the transmission route in marine animals has not been elucidated, mammals has long been considered as the dead-end host of JEV and WEV, indicating possible viral transmission by mosquitoes in marine animals [21]. The detection of JEV and WEV in infected marine animals suggest possible contamination of the water environment by infected hosts, in addition to domestic sources. While our study determined the viral survival rates and RNA detection, the focus of the study was on the feasibility of using water samples for the detection of flaviviruses. Our study showed that all flavivirus RNA tested was detectable among all water samples over the testing period (Day 0, 4 and 7) at both temperatures 4 °C and 25 °C. However, retention of virus infectivity significantly varied under the tested temperatures and incubation time. The viral RNA copy number maintained high stability at 4 °C indicating that lower temperatures had little influence on flavivirus degradation. However, at room temperature, the RNA degradation is increased resulting in virus inactivation. The findings align with previous studies which demonstrated that thermal stability of RNA structures and temperature dependence of RNA degradation [23]. Meanwhile, the infectivity of the viruses decreased rapidly while experiencing fluctuations among the water samples at 25 °C compared to 4 °C, emphasizing the ability of viral RNA to remain intact while the infectivity rate is extremely reduced at higher temperatures [23, 24]. Similarly, this can be evident by the longer half-life of infectious virus at 4 °C which preserves virus infectivity at cooler environments. Viruses cannot replicate outside a host and survival periods differ based on the environmental conditions, however, low temperatures and the presence of organic matter in water, may keep the virus particles intact and extend their survival period [25]. The findings of previous studies, demonstrated ZIKV survival in sewage samples up to 8 days [26]. However, this study focused on constant temperature of water at high virus titers, and further studies that better reflect the environmental conditions is needed.

Notably, the sea water expressed the highest decline in stability and infectivity of flaviviruses across all aquatic samples. Our results demonstrated that mosquito-borne flaviviruses were less stable in sea water, which had the highest salinity in comparison to other water samples tested in this study (Supplementary Table 1). Previous studies had demonstrated that viral activity had an inverse relationship with salinity, regardless of the pH value [27]. In this context, the inactivation of influenza A virus is associated with NaCl concentration, suggesting that salt concentration has an important effect on virus stability and infectivity [28]. Unlike other water samples, all spiked virus samples in the tap water consistently demonstrated the least reduction in RNA stability and virus infectivity at both temperatures 4 °C and 25 °C. The tap water samples used in this study complies with the international drinkable water criteria and is multi-sourced supply consisting of a blend of surface water and groundwater [29] and has been reported to possess the properties that collectively eliminate variables which could negatively affect virus stability and infectivity such as lower contaminants and lower pH levels [30]. While further studies are needed to determine the association of virus stability in tap water, the stability could be associated with lower contaminants in the tap water sample.

Dengue has remained a public health threat in the tropics for the past few decades. However, the emergence of dengue in the sub-Saharan regions and the Middle East, there remains a need to determine virus viability and detection rates at different temperature ranges. In this context, we tested samples at a lower end of the spectrum, 4 °C, middle, 25 °C and at 37 °C. Based on the emergence of dengue in Oman, UAE and other regions in the Middle East, an additional experiment was conducted to stimulate water temperatures during the hottest season, summer, in Oman. Tropical rivers typically exhibit higher temperatures than those in temperate regions, the highest recorded temperature of the river water source was 35.7 °C according to the hydrological studies and aligning with that of a previous study, to assess pathogenic survival rate in river water [31, 32]. Hence, 37 °C was used as a reference point in the study. Also, research on viral RNA stability in water showed different outcomes based on the conditions. For example, at 4 °C and 25 °C in dechlorinated water, F-specific RNA coliphages outlived feline calicivirus, but at 37 °C, survival rates were at similar levels [33]. This implies that a temperature of 37 °C is essential for comprehending viral dynamics in water with tropical-like conditions. Corresponding to the collected water data from Oman and considering the physiological temperature, the temperature was set to 37 °C over the course of 49 days. At 37 °C, the stability of the viral RNA decreased gradually, and no infectious particles were detected after Day 4. During the prolonged incubation period, DENV-1 viral RNA remained stable, but the virus infectivity was significantly reduced by Day 14, when vRNA became undetectable at cooler temperatures except tap water. Similarly, the infectivity of DENV-1 was undetectable in almost all aquatic environments from Day 4 onwards at 25 °C and 37 °C. Therefore, at lower temperatures the stability and infectivity of flaviviruses are highly preserved over a longer period. As previously demonstrated, the transmission of DENV is highly impacted by the seasonal patterns in Oman; the transmission increases in the winter and autumn which provides the ideal environmental conditions for mosquito breeding [10]. This can be further confirmed by our current findings on the RNA stability and infectivity of DENV. Further studies are however needed to determine virus stability at temperature conditions that reflect that of natural environment.

A recent study proved that flaviviruses can be vertically transmitted by passing the virus to mosquito larvae and pupae. This can initiate the horizontal transmission of the infection cycle, implying that certain levels of DENV viral RNA can be disseminated by Aedes *aegypti* mosquito into the aquatic environment [34, 35]. It has been reported that WNV in excreta are infectious to larval mosquitoes, especially the pupae. Therefore, the WNV can be maintained within mosquito populations, and the infectious virus and viral RNA were detected in the excreta of infected mosquitoes [36]. Infectious ZIKV strains have been isolated and cultured from human urine samples, suggesting that ZIKV in contaminated aquatic environments can be transmitted by breeding mosquitoes, and ZIKV has been transmitted between AG6 mice and mosquitoes breeding through the sewage containing infective virus particles [25]. DENV transmission is known to occur both vertically and sexually among mosquitoes, which is believed to contribute to the persistence of DENV in the environment. Both *Aedes aegypti* and *Aedes albopictus* mosquitoes have been found to be susceptible to three serotypes of DENV (DENV-2, -3, and -4), and DENV can grow to high titers in larvae [37]. As flavivirus vectors breed in stagnant waters [38], this indicates that dengue can spread among larva. These findings altogether raise the possibility that the infected developing larvae may passively contribute certain levels of viral RNA and the persistence of dengue in the aquatic environments. While our results in this study demonstrated that flavivirus RNA can be detected in various aquatic environment, further studies would be required to determine the amount of infected larvae or mosquitoes in association with detectable levels of viral RNA.

## Conclusions

The results of this study highlights the potential of flavivirus surveillance in aquatic environments, especially in tropical regions. Our study also indicates that DENV, JEV, YFV and ZIKV can persist in aquatic environments with the exception of sea water. However, despite the presence of viral RNA in the aquatic samples, the rapid loss of the virus infectivity at higher temperatures suggests a lower risk of flavivirus transmission through the waterborne route depending on the environmental conditions. Further research is crucial to understand the viral particle stability and infectivity at different environments by determining viral survival rate associated with geo-temporal factors that will contribute to designing more effective surveillance systems.

## Supplementary Information

Additional file 1.

## Data Availability

No datasets were generated or analyzed during the current study.
