# Supplementary material for: Assessment of flavivirus RNA stability and infectivity in various water environments
Source: Trop Med Health. 2025 Jan 24;53:11. doi: 10.1186/s41182-025-00686-9 (PMC11763117; doi:10.1186/s41182-025-00686-9)
Supplement: Supplementary file 1 — Additional file 1. [file 41182_2025_686_MOESM1_ESM.docx]

**SUPPLEMENTARY TABLE 1**. Physiochemical properties of samples used in this study.

| Sample^1^ | pH | EC  (µS/cm) | TDS  (ppm) | Salt  (ppm) | S.G | ORP  (mV) |
| --- | --- | --- | --- | --- | --- | --- |
| Tap water | 7.55  ±0.05^2^ | 284.33  ±1.15 | 141.67  ±0.58 | 142.00  ±1.00 | 1.00  ±0.00 | 503.33  ±10.26 |
| Well water | 7.75  ±0.05 | 293.67  ±2.89 | 146.67  ±1.15 | 146.67  ±1.15 | 1.00  ±0.00 | 356.00  ±6.56 |
| River water | 8.69  ±0.04 | 287.67  ±2.31 | 144.00  ±0.00 | 144.33  ±0.58 | 1.00  ±0.00 | 211.67  ±1.15 |
| Sea water | 7.95  ±0.08 | 21970  ±60 | 11000  ±0.00 | 12430  ±120 | 1.01  ±0.00 | 292.67  ±4.51 |
| EMEM | 7.36  ±0.01 | 14370  ±60 | 7193.33  ±5.77 | 7930.00  ±17.32 | 1.01  ±0.00 | 286.67  ±3.51 |

^1^ Water samples were collected from various locations in Tokyo, Japan.

^2^ Physiochemical properties are shown as mean ± standard deviation. Experiments were performed in triplicates.
